# The First Glimpse of *Streptocarpus ionanthus* (Gesneriaceae) Phylogenomics: Analysis of Five Subspecies’ Chloroplast Genomes

**DOI:** 10.3390/plants9040456

**Published:** 2020-04-04

**Authors:** Cornelius M. Kyalo, Zhi-Zhong Li, Elijah M. Mkala, Itambo Malombe, Guang-Wan Hu, Qing-Feng Wang

**Affiliations:** 1 Key Laboratory of Plant Germplasm Enhancement and Specialty Agriculture, Wuhan Botanical Garden, Chinese Academy of Sciences, Wuhan 430074, China; cmulili90@gmail.com (C.M.K.); mkala@wbgcas.cn (E.M.M.); qfwang@wbgcas.cn (Q.-F.W.); 2 University of Chinese Academy of Sciences, Beijing 100049, China; lizhizhong@wbgcas.cn; 3 Sino-Africa Joint Research Center, Chinese Academy of Sciences, Wuhan 430074, China; 4 East African Herbarium, National Museums of Kenya, P.O. Box 45166-00100 Nairobi, Kenya; imalombe@gmail.com

**Keywords:** *Streptocarpus ionanthus*, section *Saintpaulia*, divergence hotspots, phylogeny, polymorphism, simple sequence repeats (SSRs), genome structure

## Abstract

*Streptocarpus ionanthus* (Gesneriaceae) comprise nine herbaceous subspecies, endemic to Kenya and Tanzania. The evolution of *Str. ionanthus* is perceived as complex due to morphological heterogeneity and unresolved phylogenetic relationships. Our study seeks to understand the molecular variation within *Str. ionanthus* using a phylogenomic approach. We sequence the chloroplast genomes of five subspecies of *Str. ionanthus*, compare their structural features and identify divergent regions. The five genomes are identical, with a conserved structure, a narrow size range (170 base pairs (bp)) and 115 unique genes (80 protein-coding, 31 tRNAs and 4 rRNAs). Genome alignment exhibits high synteny while the number of Simple Sequence Repeats (SSRs) are observed to be low (varying from 37 to 41), indicating high similarity. We identify ten divergent regions, including five variable regions (*psb*M, *rps*3, *atp*F*-atp*H, *psb*C*-psb*Z and *psa*A*-ycf*3) and five genes with a high number of polymorphic sites (*rps*16, *rpo*C2, *rpo*B, *ycf*1 and *ndh*A) which could be investigated further for phylogenetic utility in *Str. ionanthus.* Phylogenomic analyses here exhibit low polymorphism within *Str. ionanthus* and poor phylogenetic separation, which might be attributed to recent divergence. The complete chloroplast genome sequence data concerning the five subspecies provides genomic resources which can be expanded for future elucidation of *Str. ionanthus* phylogenetic relationships.

## 1. Introduction

*Streptocarpus ionanthus* (H. Wendl.) Christenhusz (Gesneriaceae) is a complex species, within *Str.* section *Saintpaulia* [1], characterized by morphological heterogeneity among the constituent nine subspecies. The species is largely traded across America and Europe for its ornamental value, as crosses among the subspecies have produced extensive flower colors [2] after a century of intensive breeding [3]. The distribution of *Str. ionanthus* extends from coastal Kenya to Tanga and Morogoro regions in Tanzania [4], regions experiencing habitat degradation due to both human and climate change effects [5]. *Str. ionanthus* is the only member of sect. *Saintpaulia* which has been recorded to occur in exposed habitats outside dense and closed canopy forests, environs which are prone to human activities. This has led to diminishing of population sizes and even the disappearance of most populations, leading to endangered status in taxa such as *Str. ionanthus* subspecies *rupicola*, *velutinus*, *grandifolius* and *orbicularis* according to the International Union for Conservation of Nature (IUCN) Red List of Threatened Species [6].

The former genus *Saintpaulia* H. Wendl. has attracted research attention over the last two decades, witnessing inconsistent taxon classification for both molecular and morphological studies. Previous phylogenetic studies have applied few markers, both nuclear [7,8,9] and chloroplast regions [1], aiming to understand the evolutionary relationship, but without satisfactory findings. The Internal Transcribed Spacer (ITS) phylogeny [7], for instance, could not separate taxa of the *Str. ionanthus* group. Further, the 5S nuclear ribosomal DNA non-transcribed spacer (5S-NTS) data [9] displayed mixed phylogenetic signals, especially for the lower taxonomic units of *Str. ionanthus.* These observations challenge the narrow species concept used by Burtt [10,11] to describe most Usambara and adjacent populations as species, although this concept was reviewed and updated by Darbyshire [12]. Although the chloroplast phylogeny [1] also observed similar taxonomic challenges in *Str. ionanthus*, this study made tremendous progress in *Saintpaulia* research by recognizing ten species under sect. *Saintpaulia*.

Recently, the amount of sequence data available has increased due to the advent of Next-Generation Sequencing (NGS) and relatively lower sequencing costs [13,14]. Presently, more than 4000 complete chloroplast genome sequences are available in the National Center for Biotechnology Information (NCBI) database (https://www.ncbi.nlm.nih.gov/genomes). The chloroplast sequence is characterized by uniparental inheritance and a substitution rate approximately half that of the nuclear genome [15]. This low nucleotide substitution, coupled with a maternal inheritance and non-recombinant nature, makes plant chloroplast genomes appreciated sources of molecular markers for evolutionary studies [16]. Further, chloroplast genomes have demonstrated to be effective in resolving tough phylogenetic relationships, especially at lower taxonomic levels of recent divergence [17,18].

The poor resolutions and low bootstrap support values observed previously in *Str. ionanthus* suggests a case of a recently divergent group which needs to be investigated with methods other than gene-based approaches. Understanding the evolutionary relationship among such recently divergent lineages has been achieved using massive DNA data as opposed to a few genes [19,20]. Thus, chloroplast genomic analyses of *Str. ionanthus* constituent taxa could elucidate its evolutionary relationship. Presently, only one chloroplast genome exists in sect. *Saintpaulia* and none in *Str. ionanthus.* Here, we sequence chloroplast genomes of five subspecies of *Str. ionanthus* aimed at (1) reporting the annotation and sequence variation, (2) screening for divergence hotspots, and (3) providing new genomic resources for future *Str. ionanthus* research.

## 2. Results

### 2.1. Overall Features of Str. ionanthus Chloroplast Genome

**Figure 1:**
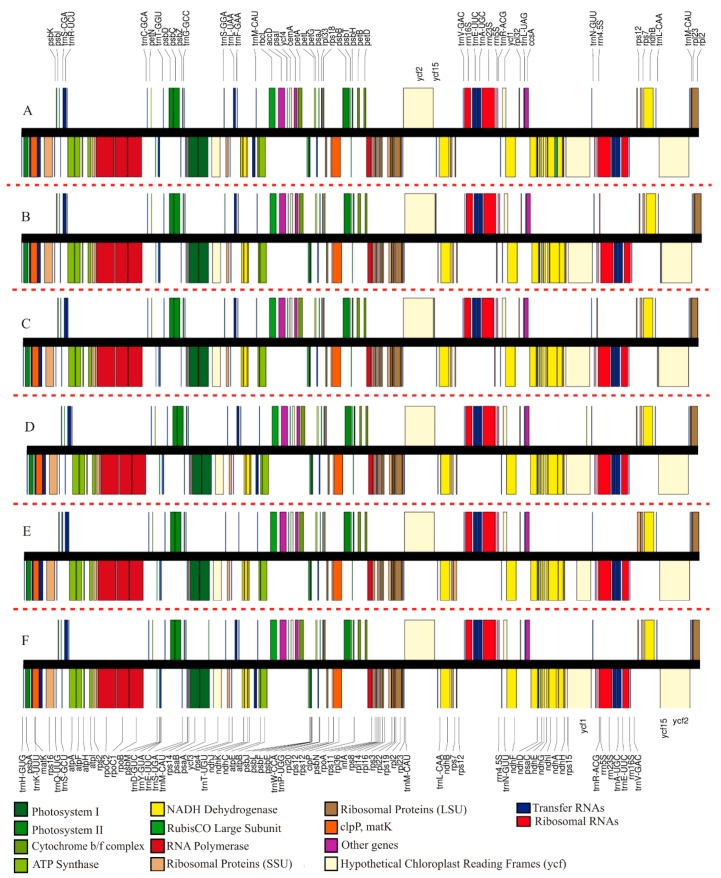
Linear chloroplast genome maps of six taxa of sect. *Saintpaulia* ((**A**) *Str. teitensis*; (**B**) subsp. *velutinus*; (**C**) subsp. *grandifolius*; (**D**) subsp. *orbicularis*; (**E**) subsp. *grotei* and (**F**) subsp. *rupicola*). The genes above the black line (names on top of the figure) represent clockwise transcription while genes below (names at the bottom) are transcribed counter-clockwise. Genes of different functional categories are colored according to the legend at the bottom.

A linear visualization of six sect. *Saintpaulia* taxa is presented in Figure 1. The chloroplast genome sizes within *Str. ionanthus* extended from 153,208 base pairs (bp) (*Str. ionanthus* subsp. *grandifolius*) to 153,377 bp (*Str. ionanthus* subsp. o*rbicularis*) (Table 1), exhibiting closeness to *Str. teitensis* with 153,207 bp [21]. Similar to other angiosperms, the five chloroplast genomes exhibited a four-partitioned structure made of a large single copy region (LSC), two inverted repeat regions (IRA and IRB) and a small single copy region (SSC) located between the Inverted Repeat (IR) regions. The length of the LSC region ranged from 84,010 bp (*Str. ionanthus* subsp. *grotei*) to 84,115 bp (*Str. ionanthus* subsp. *velutinus*), while the SSC size exhibited a variation from 18,316 bp (*Str. ionanthus* subsp. *grotei*) to 18,332 bp in two subspecies (*Str. ionanthus* subsp. *velutinus* and *Str. ionanthus* subsp. *grandifolius*). The IR regions varied from 25,431 bp (*Str. ionanthus* subsp. *velutinus* and *Str. ionanthus* subsp. *grandifolius*) to 25,464 bp (*Str. ionanthus* subsp. *orbicularis*) (Table 1). The five genomes had a total of 115 unique genes (each) including 80 protein-coding (PCGs), four ribosomal RNA (rRNAs) and 31 transfer RNA genes (tRNAs) (outlined in Table 2).

All five subspecies exhibited a duplication of 18 genes, including seven tRNAs (*trn*M-CAU, *trn*L-CAA, *trn*V-GAC, *trn*E-UUC, *trn*A-UGC, *trn*R-ACG and *trn*N-GUU), the four rRNAs, and seven PCGs (*rpl*2, *rpl*23, *ycf*2, *ycf*15, *ndh*B, *rps*7 and *rps*12). A total of 15 genes (*ndh*A, *ndh*B, *pet*B, *pet*D, *rpl*2, *rpl*16, *rpo*C1, *rps*12, *rps*16, *trn*A-UGC, *trn*G-UCC, *trn*I-GAU, *trn*K-UUU, *trn*L-UAA, and *trn*V-UAC) contained a single intron, whereas two genes (*clp*P and *ycf*3) contained two introns each. Compared to the congeneric *Str. teitensis* [21], the six genomes generally had a high similarity, although *Str. teitensis* had 114 genes due to the absence of the gene *ycf*15.

### 2.2. Comparison of Chloroplast Genome Structure in Sect. Saintpaulia

**Figure 2:**
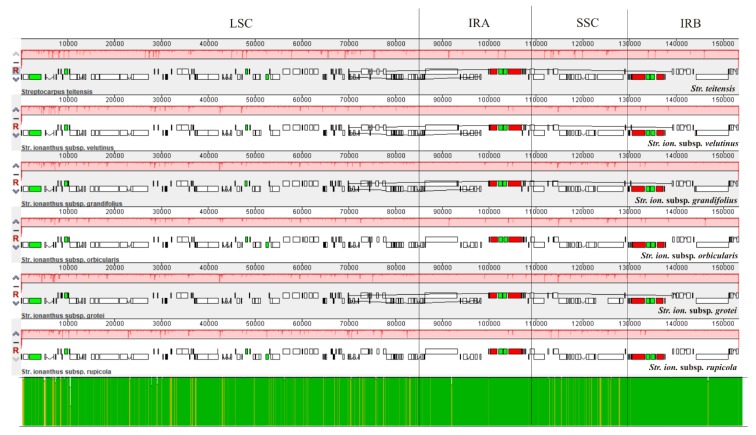
Multiple genome alignment of six sect. *Saintpaulia* taxa. The red bars represent sequence similarity among different genomes. The bottom bar is a visualization of the sequences’ consensus identities with the green color symbolizing homology while the yellow vertical lines signify variation spots. LSC: Large Single Copy region; IRA: Inverted Repeat A; SSC: Small Single Copy region and IRB: Inverted Repeat B.

**Figure 3:**
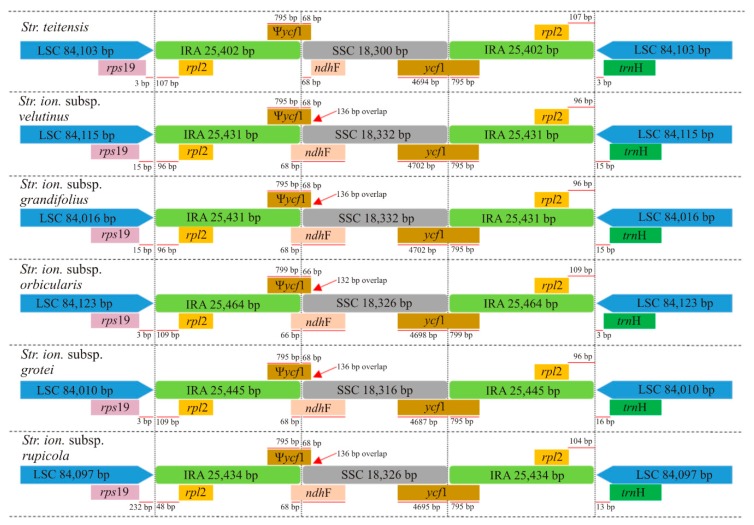
Comparison of the Inverted Repeat/ Single Copy (IR/SC) junctions’ characteristics among the six sect. *Saintpaulia* genomes. The genes below and above are transcribed in clockwise and counter-clockwise directions, respectively. The setting is not to scale.

The structural alignment in Mauve revealed one synteny block (in red) with a conserved gene order, minimal structural disparity and no rearrangements among the six genomes (Figure 2). Further, within the Large and Small single copy regions (LSC and SSC), very minor sequence variations were observed, as exhibited by the red vertical lines in the genome blocks and the yellow vertical lines in the consensus sequence identity (green block). However, the Inverted Repeat (IR) regions were relatively more conserved, as displayed by the green block. Comparison of the genes present at the Inverted Repeat/ Single Copy (IR/SC) junctions (Figure 3) revealed that the Large Single Copy/ Inverted Repeat A (LSC/IRA) junction occurred between the *rps*19 and *rpl*2 genes for all species while the IRA/SSC was characterized by an overlap of the *ycf*1*-ndh*F genes, except in *Str. teitensis* in which the genes were next to each other. Further, the Small Single Copy/ Inverted Repeat B (SSC/IRB) junction was characterized by the *ycf*1 gene while the IRB/LSC junction occurred between the genes *rpl*2 and *trn*H. The SSC/IRB junction extended into the *ycf*1 gene creating a *ycf*1 pseudogene with a conserved length (795–799 bp) in the IRA/SSC junction. To conclude, all junctions had similar genes with only slight variations in the distance between the junctions and adjacent genes.

### 2.3. Divergent Hotspots and Simple Sequence Repeats (SSRs) in Str. ionanthus

**Figure 4:**
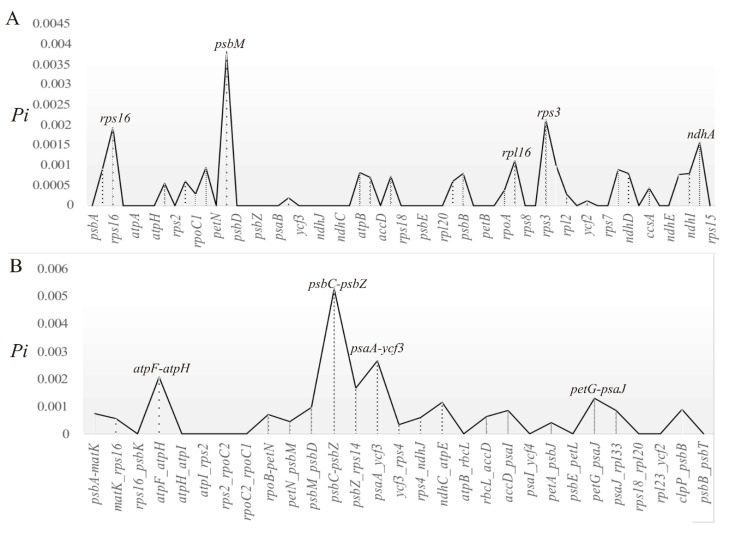
Nucleotide variability (*Pi*) values among chloroplast genomes of the five *Str. ionanthus* subspecies for (**A**) Coding sequences and (**B**) Intergenic spacer regions.

The values of nucleotide variability (*Pi*) across the analyzed coding and intergenic sequences of the five subspecies ranged from 0 (majority) to 0.00526 (*psb*C*-psb*Z) (Figure 4), with a low average value (*Pi =* 0.00050). The total alignment file was 153,533 bp, with 152,813 sites (99.53%) being monomorphic while only 184 sites were polymorphic of which subsp. *rupicola* had the majority of Insertion and Deletions (InDels). Twenty-six Protein-Coding genes (PCGs) were observed to contain polymorphic sites, with only five genes having more than five sites (*rps*16*_*9, *rpo*C2*_*6, *rpo*B*_*6, *ycf*1*_*8 and *ndh*A*_*7). The majority of the polymorphic sites (169) were singleton variable sites and there were only 15 parsimony informative sites, representing a relatively low variation among the subspecies. Despite the low variation, ten regions exhibited some polymorphism (hereafter termed as divergence hotspots), including five regions with *Pi* > 0.002 (*psb*C*-psb*Z, *psb*M, *psa*A*-ycf*3, *rps*3 and *atp*F*-atp*H) and five PCGs with more than five polymorphic sites.

**Figure 5:**
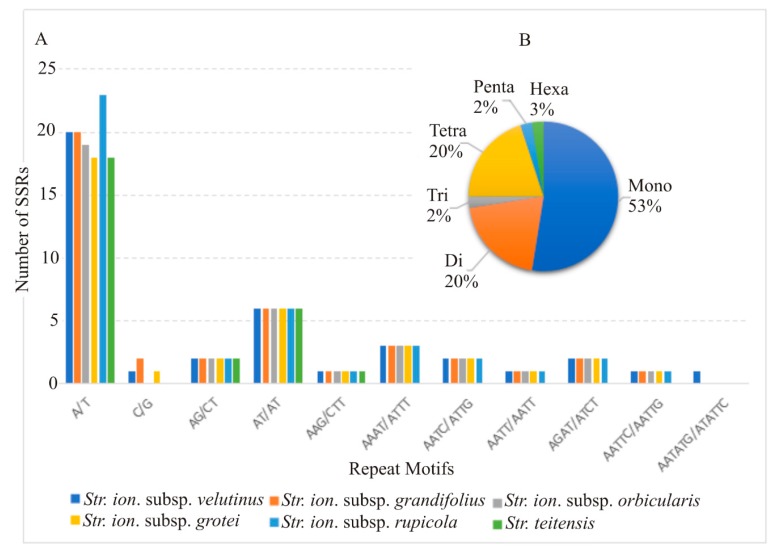
Simple Sequence Repeats (SSRs) in six sect. *Saintpaulia* chloroplast genomes. (**A**) Number of identified SSRs at different repeat motifs, (**B**) Percentage contribution of each repeat type.

SSRs range from mono-to hexa-nucleotide repeat units which exhibit polymorphism even within one species and occur widely in plant genomes. Sect. *Saintpaulia* cp genomes exhibited small variation in the number of SSRs with two subspecies (*Str. ionanthus* subsp. *velutinus* and *Str. ionanthus* subsp. *grandifolius*) having 40 SSRs, two subspecies (*Str. ionanthus* subsp. *orbicularis* and *Str. ionanthus* subsp. *grotei*) having 37 SSRs while *Str. ionanthus* subsp. *rupicola* and *Str. teitensis* have 41 and 28 SSRs, respectively (Figure 5A). Further, the mononucleotides dominated, followed by both dinucleotides and tetranucleotides. while the Trinucleotides (2%), pentanucleotides (2%) and hexanucleotides (3%) were the minority (Figure 5B). The intergenic regions housed the majority (55–60%) of the SSRs, while the intron and coding sequences accounted for the approximately 40% remaining. The coding genes having SSRs included *rpo*C2, *psb*C, *atp*B, *rpl*22, *ndh*A and *ycf*1.

### 2.4. Phylogenetic Analysis

**Figure 6:**
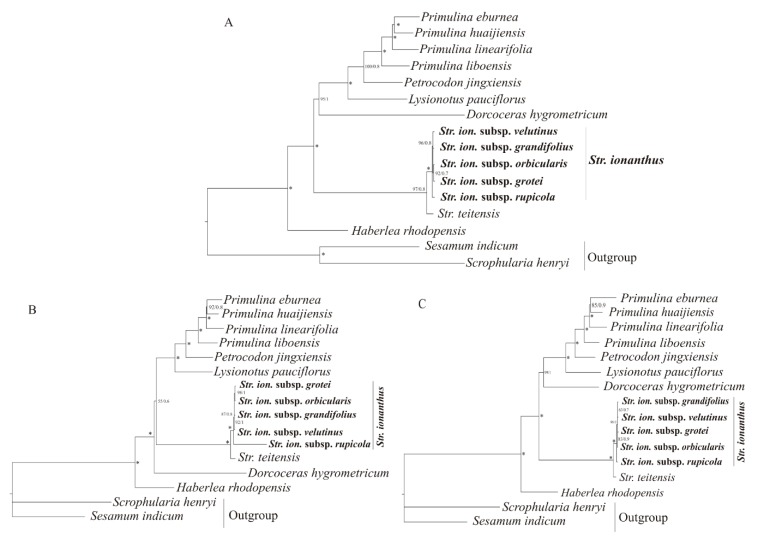
The phylogenetic relationship within five *Str. ionanthus* subspecies and the relationship with other Gesneriaceae based on (**A**) complete genome sequence and (**B**) coding genes and (**C**) intergenic regions. The bootstrap support values are given for both Maximum Likelihood (ML) and Bayesian Inference (BI) trees (ML/BI) and * denote maximum support values for both ML/BI.

The phylogenetic relationship presented identical topology for both Maximum Likelihood (ML) and Bayesian Inference (BI) tree approaches, as shown in Figure 6. Regarding Gesneriaceae, *Streptocarpus* was closer to *Dorcoceras* and *Lysionotus*, while *Petrocodon* was closer to *Primulina* and *Haberlea* was distantly placed. The four species of *Primulina* displayed a close relationship with each other while *Str. ionanthus* genomes used here exhibited monophyly from *Str. teitensis*. Concerning the *Str. ionanthus*, subspecies *rupicola* exhibited a relative distinction from the other four, subsp. *velutinus* and subsp. *grandifolius* grouped together and were sistered to the grouping of subsp. *orbicularis* and subsp. *grotei*. Our data report a poor phylogenetic structure within *Str. ionanthus*, findings in line with some previous studies.

## 3. Discussion

### 3.1. Analysis of Genome Features

During this study, we sequence and compare the major features of five *Str. ionanthus* subspecies chloroplast genomes. Generally, the angiosperm chloroplast genome is considered to be conserved [15]. The *Str. ionanthus* taxa used here reveal the typical angiosperm structure with identical genes, gene order and no structural reconfigurations. The genomes exhibit a narrow size range (170 bp) and do not deviate from the first reported chloroplast genome in sect. *Saintpaulia* [21]. However, much lower size ranges have been reported in the *Hosta* (<85 bp) [22] and *Pyrus hopeiensis* (46 bp) [23] species and, thus, *Str. ionanthus* cp genomes can be termed as relatively variable. 

Seen in the chloroplast genome, the Inverted Repeat (IR) region is reported to be stable [24] with border shifts contributing to the evolution of species, including variation in genome sizes [23,25]. Our study supports this, with *Str. ionanthus* subsp. *orbicularis* having the longest IR region and also being the largest of the five genomes in terms of complete genome size. The representative *Str. ionanthus* cp genomes in this study are characterized by similar genes in the Inverted Repeat/ Single Copy (IR/SC) boundaries, with slight variations in the length flanking or drifting away from the boundaries. Nonetheless, other reported Gesneriaceae genomes vary from *Str. ionanthus* in some junctions. The Large Single Copy/ Inverted Repeat A (LSC/IRA) occurs between *rps*19–*rpl*2 in sect. *Saintpaulia* and *Harbelea* [26], *rpl*22*–rpl*2 in *Petrocodon* [27] and inside *rps19* in *Primulina* [28], *Dorcoceras* [29] and *Lysionotus* [30] genomes. Diversity within Gesneriaceae also is noted in the IRA/SSC junction with *Str. ionanthus* genomes being similar to *Petrocodon*, *Dorcoceras* and *Lysionotus*, by having an overlap of *ycf*1 and *ndh*F genes, and different from *Str. teitensis*, *Haberlea* and *Primulina* which have *ycf*1. However, the other two junctions are similar within Gesneriaceae.

Besides the similarity in the IR/SC junctions, the high genome synteny with minor variations reported in the Mauve alignment portray a conserved cp genome in *Str. ionanthus*. Accompanying the absence of observable structural variations, the minor variations exhibited by the red/yellow lines in the single copy regions could be attributed to the presence of Insertions and Deletions (InDels) in those regions, especially the non-coding regions, as reported in another study [31]. Mixed observations have been reported in angiosperm chloroplast genomes, with some exhibiting high variation and others being relatively conserved. Previous genomic analyses involving higher taxonomic ranks such as the order Dipsacales [32] or family Ranunculaceae [33] have reported substantially higher genome variations in terms of gene content, arrangement and structural rearrangements such as inversed regions. However, genomic exploration at the genera levels in *Notopterygium* [34], *Camellia* [24], *Prunus* [35], *Meconopsis* [36], just to mention a few, have demonstrated highly conserved chloroplast genomes among constituent species. Found in much lower taxonomic levels, studies involving four varieties of *Arachis hypogaea* (peanut) [31], seventeen individuals of *Jacobaea vulgaris* [37], two *Ulmus americana* (elm) genotypes among others, reveal very high cp genome similarities. Thus, the high genome similarity among *Str. ionanthus* subspecies is expected. Interestingly, some studies such as *Pyrus* cultivars [38] report a relatively high variability among low taxonomic ranks.

### 3.2. Divergence Hotspots in Str. ionanthus

Simple Sequence Repeats (SSRs) are important sources of information for genetic diversity and polymorphism testing [24] due to motif variations, a high number of repetitions, and genome-wide distribution [39]. The distribution of SSRs in cp genomes is mostly concentrated in the intergenic spacers and intron regions rather than in the genes [40]. This is the case in our study where the number of SSRs in the intergenic regions are the majority (55–60%), while the introns and coding sequences contribute approximately 20% each. Since the chloroplast is conserved in angiosperms, chloroplast SSRs are transferrable across species and genera [24] and, thus, the SSR data explored in the present study provide useful information for the design of phylogenetic markers for future use. Though the number of SSRs is low, the Adenine/ Thymine (A/T) motifs vary within *Str. ionanthus*, with the subspecies *rupicola* having the highest quantity.

The overall nucleotide variability in *Str. ionanthus* cp genomes is comparatively lower (*Pi =* 0.0006) than in some other reported taxa (*Cardiocrinum*: *Pi* = 0.003; *Papaver*: *Pi* = 0.009) [41,42], an expected result in this case of a lower taxonomic level. Insertions and Deletions (InDels) are known to contribute the most microstructural variation in chloroplast genomes [23]. Here, InDels are attributed to the polymorphic sites detected in the ten divergent regions (*psb*C*-psb*Z, *psa*A*-ycf*3, *atp*F*-atp*H, *psb*M, *rps*3, *rps*16, *rpo*C2, *rpo*B, *ycf*1 and *ndh*A). Although these divergence regions were discovered in *Str. ionanthus*, the majority of them occur in *Str. ionanthus* subsp. *rupicola* which limits their ability to separate the Usambara taxa. However, this result should be interpreted with caution and more sampling could reveal interesting details about the variation of these genome regions. The extremely high polymorphism of *Str. ionanthus* subsp. *rupicola* may be partly due to long-term isolation of the subspecies from the others.

The observed low variability means that a majority of the genome regions are of limited capacity for phylogenetic studies, thus previously applied chloroplast regions could not resolve *Str. ionanthus* classification. The coding and non-coding sequences have varied substitution rates [23]. Non- coding regions are less controlled by function and have relatively higher nucleotide substitution rates causing rapid evolution, thus, are more preferred for phylogenetic studies in lower taxonomic level taxa [23,43]. Similar to reports in most angiosperms [44], the intergenic regions in *Str. ionanthus* exhibit higher nucleotide diversity than the coding regions, with the most variable region being *psb*C*-psb*Z. Studies in higher plants have reported a high variability of *mat*K, *rps*16 and *rbc*L [45] and other non-coding regions [46,47], thus are proposed for phylogenetic studies. Analysis of three *Pyrus* specie chloroplast genomes [48] identify four divergence hotspots (*pet*N*-psb*M, *psb*M*-trn*D, *rps*4*-trn*T*-trn*L, and *psa*I*-ycf*4) having an average variation of *Pi =* 0.00054. However, in our study, most of these regions exhibit very low or no polymorphism. The divergence hotspots detected here could be tested further for utility in the phylogenetic analyses using all subspecies and more samples. Our results are valuable for future studies on estimating the variation within *Str. ionanthus.*

### 3.3. Phylogenetic Relationship within Str. ionanthus

The relative stability of molecular data makes them useful in estimating phylogenetic relationships among species [24]. Despite making great milestones in sect. *Saintpaulia* phylogenetics, previous phylogenetic studies [1,7] were unable to obtain a high-resolution and strongly-supported phylogeny in *Str. ionanthus*, although these studies applied few markers. Here, we report the first genome-scale phylogenetic analysis in sect. *Saintpaulia* by comparing the phylogenetic relationship among the six sequenced taxa and within Gesneriaceae. However, we admit the fact that our study might not make entirely conclusive remarks on *Str. ionanthus* phylogeny due to the limited number of genomes. Nevertheless, our observations are consistent with most earlier studies and sets the blueprint for future phylogenomic analyses in understanding *Str. ionanthus*.

Rapid evolution leads to poorly-resolved phylogenies [49] and produce short branches with little nucleotide polymorphism observed, which imply a recent divergence. Previously, molecular dating studies on *Str. ionanthus* using both nuclear [4] and chloroplast (Kyalo, unpublished) genes have demonstrated a case of recent diversification (<2 million years ago). This could explain the short branches observed in our study. However, the high bootstrap support in the present study shows the ability of complete genomes to improve the phylogenetic resolutions in plant evolution [50,51] and adding more genomes to this complex can produce a conclusive phylogeny of *Str. ionanthus*. *Str. ionanthus* subsp. *rupicola* is presented as distinct from the other four subspecies in all datasets used here, although this is not a new finding as similar outcomes have been reported in previous studies. This can be geographically explained in that *Str. ionanthus* subsp. *rupicola* occurs in Kenya while the other four subspecies are distributed in the Usambara mountains (Tanzania).

## 4. Materials and Methods

### 4.1. Sampling, Laboratory Experiments and Sequencing

**Figure 7:**
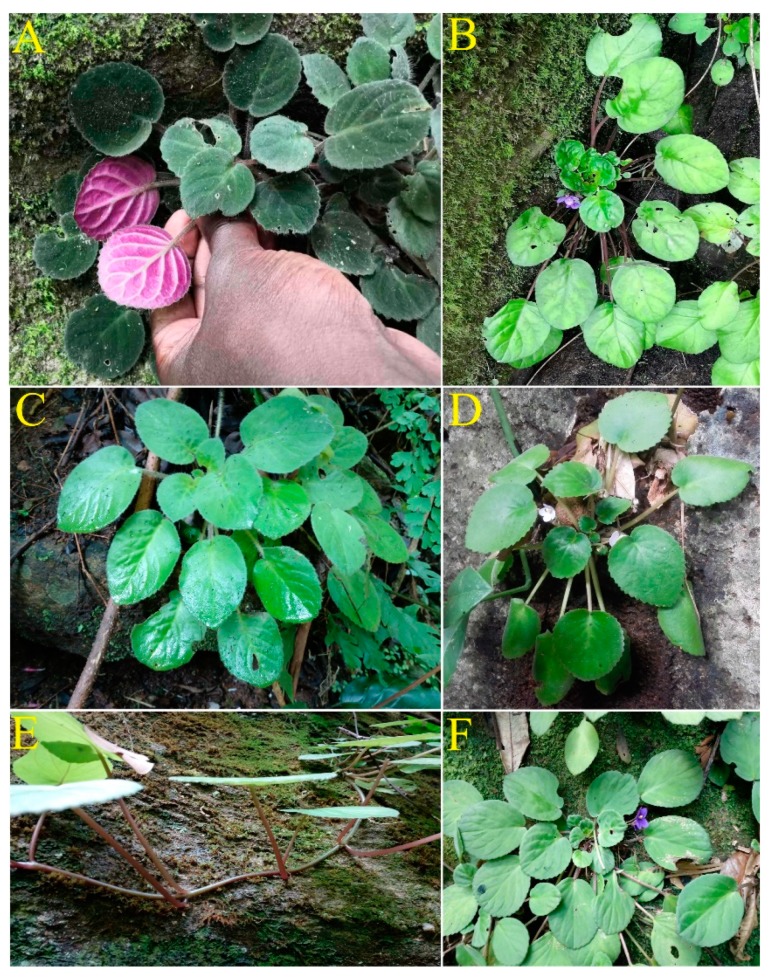
Morphological heterogeneity in *Streptocarpus ionanthus*; (**A**) *Str. ionanthus* subsp. *velutinus*, (**B**) *Str. ionanthus* subsp. *orbicularis*, (**C**) *Str. ionanthus* subsp. *grandifolius*, (**D**) *Str. ionanthus* subsp. *rupicola*, (**E**) *Str. ionanthus* subsp. *grotei* (trailing habit) and (**F**) *Str. ionanthus* subsp. *grotei* (rosulate habit).

We collected leaf samples of five subspecies of *Str. ionanthus* (illustrated in Figure 7) from the Usambara mountains (Tanzania) and Kilifi (Kenya) based on the countries’ laws governing collection and exportation of biological samples for research purposes. The samples were dried in silica gel for further laboratory experiments. Genomic DNA was extracted from each leaf sample using Plant DNAzol Reagent (Life Feng, Shanghai) following the manufacturer’s instructions. Sequencing was done using the Illumina HiSeq 2000 platform from the Tsingke company (Wuhan, China), obtaining raw reads.

### 4.2. Assembly and Gene Annotation

Filtration was performed on the raw Illumina reads using an NGS QC tool kit [52] to eliminate low-quality reads. The resultant clean reads of the five subspecies were mapped alongside the reference chloroplast genome of *Str. teitensis* (GenBank Accession: MF596485) using the program Bowtie ver. 2.2.6 [53], following the default settings. Assembly of the chloroplast genome reads into contigs was done by Velvet ver. 1.2.10 [54] set at k-mer of 75, 85, 95 and 105. The verified contigs were subjected to BLAST and library searches and connected into complete genomes in SPAdes ver. 3.10.1 [55] with parameters set to default. The products of the Assembly were visualized and manually corrected in Bandage ver. 8.0 [56].

Genome annotation was done using the GeSeq application [57], an online tool in the Chlorobox database (https://chlorobox.mpimp-golm.mpg.de/index.html), combined with manual corrections to confirm the start and stop codons. The program tRNAscan-SE ver. 1.21 [58] was used to verify the identified tRNA genes. The genome maps were developed in the Organellar Genome Draw program (OGDRAW) ver. 1.3.1 [59]. Classification of the annotated genes according to functionality was conducted with reference to the online CpBase database (https://rocaplab.ocean.washington.edu/tools/cpbase/). The annotated genomes were submitted to the National Center for Biotechnology Information (NCBI) GenBank database (Accession numbers provided in Table 1).

### 4.3. Genome Comparison

Genome features such as the expansion or contraction in the Inverted Repeat/ Single Copy (IR/SC) junctions, structural re-organization and the loss or pseudogenization of genes have been used in previous studies to inform an evolutionary history of species [60]. Comparison of these features was performed among the available six sect. *Saintpaulia* cp genomes (Table 1). The IR/SC junctions were analyzed to detect possible expansion or contraction through identification of the genes present or adjacent to the junctions. To determine the gene order and identify possible structural re-arrangements among the six cp genomes, multiple alignment of the genomes was done using the program Mauve [61]. During this analysis, progressiveMauve was set as the alignment algorithm, full alignment was automatically calculated, and the genomes were assumed to be non-collinear.

### 4.4. Identification of Divergent Hotspots and Simple Sequence Repeats (SSRs)

Intraspecific variations within the five *Str. ionanthus* genomes were identified using nucleotide diversity values (*Pi*) of the aligned sequence, executed in DNA Sequence Polymorphism (DnaSP) ver. 6.0 [62]. The settings for DNA polymorphism analysis were a window length of 800 bp and the step size set to 200 bp. Further, this analysis narrowed to check the variability of coding genes and the intergenic regions. The results indicated similar variable peaks and, thus, the graphs for coding genes and intergenic regions are presented here. We also estimated the number of polymorphic sites in each of the 62 protein coding genes with DnaSP ver. 6.0. Mutations are key variants which can lead to polymorphism among taxa. Here, mutations among the five genomes of *Str. ionanthus* were evaluated by analyzing the number of Insertions and Deletions (InDels) using DnaSP and, eventually, confirmed manually from the aligned sequences.

Simple Sequence Repeats (SSRs) were identified from the six sect. *Saintpaulia* genomes using MISA (Microsatellite Identification tool) on the web version [63]. The selection criteria were minimum repeat thresholds of 10, 5, 4, 3, 3 and 3 for mononucleotide, dinucleotide, trinucleotide, tetranucleotide, pentanucleotide and hexanucleotide repeats, respectively.

### 4.5. Phylogenetic Analysis

Since the focus of this study was on understanding *Str. ionanthus*, the phylogenetic relationship was explored at the family level using the other nine Gesneriaceae chloroplast genomes and two outgroups already deposited in the National Center for Biotechnology Information (NCBI) (Table S1). We applied both Maximum Likelihood (ML) and Bayesian Inference (BI) approaches using three datasets—the complete genome sequences, 62 protein coding gene sequences and 30 intergenic spacer sequences. The sequences were aligned in Multiple Alignment using Fast Fourier Transform (MAFFT) [64]. The ML analysis was implemented in IQ-TREE ver. 1.6.1 [65], with the substitution model chosen by ModelFinder [66]. Based on the Bayesian Information Criterion (BIC), the best-fitting models for the ML analyses were TVM + F + R2 for both complete genomes and intergenic spacers, and GTR + F + R2 for coding genes. The branch supports were estimated with 5000 bootstrap replicates and 1000 maximum iterations via the UltraFast Bootstrap approximation [67]. The BI analysis was conducted in MrBayes ver. 3.2.6 [68] by running four chains for two million generations. Sampling of the trees was done every 1000 generations, with the first 25% of the sampling being discarded as burn-in while the remaining were used to construct a 50% majority rule consensus tree. The best-fitting substitution models were GTR + F + I + G4 for complete genomes, intergenic spacers and GTR + F + G4 coding genes, respectively. The output trees were visualized in FigTree ver. 1.4.2 (http://tree.bio.ed.ac.uk/software/figtree/).

## 5. Perspectives on *Streptocarpus ionanthus* Research

It is undoubtedly crucial to expound on the genetic relationships within *Str. ionanthus* to understand the species evolution and inform development of horticultural cultivars. We performed comparative analysis to estimate the level of variation in gene arrangement, mutation spots, repeat sequences and phylogenetic relationships among five *Str. ionanthus* taxa and other Gesneriaceae. The majority of the phylogenetic markers developed as barcodes for angiosperm classification have proven useful in resolving phylogenetic relationships in higher taxonomic levels but are rarely informative at lower levels. Seen in *Str. ionanthus*, the nine subspecies exhibited poor resolutions and mixed signals in previous phylogenies which used few molecular markers. No clear phylogenetic distinction has been reported among the subspecies, except subspecies *rupicola* which exhibits a clear monophyly within the complex. This implies a case of recent divergence in *Str. ionanthus*, especially in the Usambara mountains taxa. To the best of our knowledge, this study presents the first genome-scale analysis in the group and the findings exhibit a close phylogenetic relationship and low sequence variation among the five subspecies investigated. However, our study identified some divergent hotspots which could be explored for polymorphism with more sampling and applied to shed more light on the evolution of *Str. ionanthus*. Our work can be a blueprint for progressive molecular research in *Str. ionanthus*, especially phylogenomic analysis which should incorporate the entire species’ taxon representation and increased sampling for each taxon. To conclude, this study provided a first glimpse into the evolution of *Str. ionanthus* complex using a phylogenomic approach and opened the species to more research opportunities.

## Figures and Tables

**Table 1:** Characteristics of major features of six sect. *Saintpaulia* chloroplast genomes.

Taxa	*Str. teitensis*	*Str. ionanthus* subsp. v*elutinus*	*Str. ionanthus* subsp. *grandifolius*	*Str. ionanthus* subsp. *orbicularis*	*Str. ionanthus* subsp. *grotei*	*Str. ionanthus* subsp. *rupicola*
Accession Number	MF596485	MN935472	MN935471	MN935470	MN935469	MN935473
Total size (bp)	153,207	153,307	153,208	153,377	153,215	153,290
LSC size (bp)	84,103	84,115	84,016	84,123	84,010	84,097
SSC size (bp)	18,300	18,332	18,332	18,326	18,316	18,326
IR size (bp)	25,402	25,431	25,431	25,464	25,445	25,434
Number of genes	114	115	115	115	115	115
Number of PCGs	79	80	80	80	80	80
Number of tRNAs	31	31	31	31	31	31
Number of rRNAs	4	4	4	4	4	4

LSC: Large Single Copy region; SSC: Small Single Copy region; IR: Inverted Repeat region; PCGs: Protein Coding genes; tRNAs: transfer RNA genes; rRNAs: ribosomal RNA genes.

**Table 2:** Genes present in the chloroplast genomes of five *Str. ionanthus* subspecies.

Category	Gene Names
Photosystem 1	*psa*A, *psa*B, *psa*C, *psa*I, *psa*J
Photosystem 11	*psb*A, *psb*B, *psb*C, *psb*D, *psb*E, *psb*F, *psb*H, *psb*I, *psb*J, *psb*K, *psb*L, *psb*M, *psb*N, *psb*T, *psb*Z
NADH Dehydrogenase	*ndh*A ^a^, *ndh*B ^a,c^, *ndh*C, *ndh*D, *ndh*E, *ndh*F, *ndh*G, *ndh*H, *ndh*I, *ndh*J, *ndh*K
ATP Synthase	*atp*A, *atp*B, *atp*E, *atp*F ^a^, *atp*H, *atp*I
Cytochrome b/f complex	*pet*A, *pet*B, *pet*D, *pet*G, *pet*L, *pet*N
RubisCO large subunit	*rbc*L
RNA Polymerase	*rpo*A, *rpo*B, *rpo*C1 ^a^, *rpo*C2
Ribosomal proteins (Large)	*rpl*2 ^a^, *rpl*14, *rpl*16, *rpl*20, *rpl*22, *rpl*23 ^c^, *rpl*32, *rpl*33, *rpl*36
Ribosomal proteins (Small)	*rps*2, *rps*3, *rps*4, *rps*7 ^c^, *rps*8, *rps*11, *rps*12 ^b,c,d^, *rps*14, *rps*15, *rps*16 ^a^, *rps*18, *rps*19
Ribosomal RNAs	*rrn*4.5 ^c^, *rrn*5 ^c^, *rrn*16 ^c^, *rrn*23 ^c^
Transfer RNAs	*trn*A-UGC ^a,c^, *trn*C-GCA, *trn*D-GUC, *trn*E-UUC, *trn*F-GAA, *trn*G-GCC, *trn*G-UCC, *trn*H-GUG,
	*trn*I-CAU ^c^, *trn*I-GAU ^a,c^, *trn*K-UUU, *trn*L-CAA ^c^, *trn*L-UAA ^a^, *trn*L-UAG, *trnf*M-CAU,
	*trn*N-GUU ^c^, *trn*P-UGG, *trn*Q-UUG, *trn*R-ACG ^c^, *trn*R-UCU, *trn*S-GCU, *trn*S-GGA, *trn*S-UGA, *trn*S-CGA
	*trn*T-GGU, *trn*T-UGU, *trn*V-GAC ^c^, *trn*V-UAC ^a^, *trn*W-CCA, *trn*Y-GUA, *trn*M-CAU
	*ycf*1 ^c^, *ycf*2 ^c^, *ycf*3 ^b^, *ycf*4, *ycf*15 ^a,c^
Protease	*clp*P ^b^
Maturase	*mat*K
Translational initiation factor	*inf*A
Envelope membrane protein	*cem*A
Subunit of acetyl-CoA-carboxylase	*acc*D
c-type cytochrome synthesis	*ccs*A

^a^ Gene with one intron. ^b^ Gene with two introns. ^c^ Duplicated genes in the IR regions. ^d^ Trans-splicing gene.

## Supplementary Materials

The following are available online at https://www.mdpi.com/2223-7747/9/4/456/s1, Table S1: Species used in the phylogenetic analysis.
