# Supplementary material for: The First Glimpse of Streptocarpus ionanthus (Gesneriaceae) Phylogenomics: Analysis of Five Subspecies’ Chloroplast Genomes
Source: Plants (Basel). 2020 Apr 4;9(4):456. doi: 10.3390/plants9040456 (PMC7238178; doi:10.3390/plants9040456)
Supplement: Supplementary file 1 [file plants-09-00456-s001.pdf]

Table S1. Species used in the phylogenetic analysis.

Species	Accession Number
Str.ionanthus subsp. *grotei*	MN935469
Str.ionanthus subsp. *grandifolius*	MN935471
Str.ionanthus subsp. *orbicularis*	MN935470
Str.ionanthus subsp. *rupicola*	MN935473
Str.ionanthus subsp. *velutinus*	MN935472
Str. teitensis	MF596485
Haberlea rhodopensis	NC_031852
Lysionotus pauciflorus	NC_034660
Petrocodon jingxiensis	NC_044477
Primulina eburnea	NC_036100
Primulina huaijiensis	NC_036413
Primulina liboensis	NC_036101
Primulina linearifolia	NC_036414
Dorcoceras hygrometricum	NC_016468
Scrophularia henryi	NC_036943
Sesamum indicum	JN637766
